# Preoperative Posterior Stromal Ripples as Predictive Biomarkers of Visual Recovery After Descemet Membrane Endothelial Keratoplasty

**DOI:** 10.1097/ICO.0000000000003698

**Published:** 2024-09-20

**Authors:** Mariacarmela Ventura, Matteo Airaldi, Chiara Ancona, Enrico Neri, Erika Bonacci, Emilio Pedrotti, Alfredo Borgia, Matteo Posarelli, Hannah J. Levis, Francesco Semeraro, Stephen B. Kaye, Pietro Viola, Vito Romano

**Affiliations:** * Department of Medical and Surgical Specialties, Radiological Sciences, and Public Health, Ophthalmology Clinic, University of Brescia, Brescia, Italy;; † Department of Ophthalmology, University of Verona, Verona, Italy;; ‡ Department of Molecular and Translational Medicine, University of Brescia, Brescia, Italy;; § St. Paul's Eye Unit, Royal Liverpool University Hospital, Liverpool, UK;; ¶ Department of Eye and Vision Science, Institute of Ageing and Chronic Disease, University of Liverpool, Liverpool, UK; and; ║ Department of Ophthalmology, San Bortolo Hospital, Vicenza, Italy.

**Keywords:** DMEK, posterior stromal ripples, AS-OCT, visual acuity, recovery rate, rebubbling, biomarker

## Abstract

**Purpose:**

To investigate the role of preoperative posterior stromal ripples (pre-PSR) on visual acuity recovery after Descemet membrane endothelial keratoplasty (DMEK).

**Methods:**

This is a comparative case series retrospectively analyzing patients who underwent DMEK. Electronic records and imaging of DMEK patients were reviewed. The last preoperative and first postoperative available anterior segment optical coherence tomography scans for each eye were analyzed for the presence of pre-PSR. The difference in longitudinal trends of visual acuity recovery after DMEK was assessed in eyes with and without pre-PSR. The frequency of rebubbling and measures of proportional relative risk of rebubbling were analyzed according to the presence of preoperative and postoperative PSR.

**Results:**

A total of 66 patients (71 eyes) were included. Pre-PSR were associated with lower preoperative visual acuity [0.6 (0.5) vs. 0.9 (0.6) LogMAR, *P* = 0.02] and higher central corneal thickness [613 (73.8) vs. 715.7 (129.6) micron, *P* < 0.001]. Eyes with pre-PSR had a slower visual recovery up to 3.5 months after surgery compared to eyes without pre-PSR and achieved lower final visual acuity [0.1 (0.2) vs. 0.3 (0.3) LogMAR, *P* = 0.02]. Cox proportional hazard ratios showed that postoperative PSR were associated with a greater risk of rebubbling [hazard ratio (95% CI), 7.1 (1.3, 39.5), *P* = 0.02] while pre-PSR were not.

**Conclusions:**

The presence of pre-PSR is associated with slower visual recovery and lower final visual acuity after DMEK while postoperative PSR confer a higher risk of rebubbling. PSR represent a valuable prognostic biomarker both before and after DMEK.

Descemet membrane endothelial keratoplasty (DMEK) has superseded other surgical interventions for endothelial dysfunction, thanks to the higher postoperative visual acuity, the faster speed of recovery, and the lower rejection rates.^1,2^ However, the postoperative visual recovery following DMEK can often vary, even between cases that appear clinically similar.^3,4^ Therefore, accurately predicting the final visual outcomes after DMEK remains a challenge.

Multiple studies have demonstrated that final best corrected visual acuity (BCVA) after DMEK can be influenced by preoperative, intraoperative, and postoperative factors, as well as donor and host tissue characteristics. Besides graft characteristics such as the advanced age of the donor, low endothelial cell density (ECD), and extended storage time,^5–8^ additional studies have highlighted the impact on the best corrected visual acuity of early and chronic ultrastructural changes in the cornea of the patient before surgery.^9–12^

Recently, Coco et al^13^ defined postoperative irregularities in the posterior corneal profile as posterior stromal ripples (PSR). The postoperative PSR (post-PSR) can be observed as stromal waves on anterior segment optical coherence tomography (AS-OCT). Post-PSR were found to represent a significant postoperative risk factor of graft detachment and graft rebubbling.

Given their value as a postoperative prognostic biomarker, it is reasonable to hypothesize that PSR, when already identifiable in the preoperative period (pre-PSR), might affect DMEK outcomes and carry additional risks of early postoperative complications. In this study, we investigate the impact of pre-PSR on visual acuity, speed of recovery, and risk of rebubbling after DMEK.

## MATERIALS AND METHODS

This study has been reported according to the Strengthening the Reporting of Observational Studies in Epidemiology (STROBE) checklist for cohort studies.^14^ Local institutional review board approval was obtained (protocol no. 12783), and the study adhered to the tenets of the Declaration of Helsinki. Given its retrospective nature, waiver of informed consent was granted.

### Study Design and Setting

This research was designed as a multicenter, retrospective, comparative case series. The study was conducted in accordance with the tenets of the Declaration of Helsinki. Electronic medical records of patients who underwent DMEK from May 2021 to September 2023 at the ASST Spedali Civili di Brescia, the Azienda Ospedaliera Integrata di Verona, the San Bortolo Hospital of Vicenza, and the Liverpool University Hospitals NHS Foundation Trust were reviewed to identify possible participants.

### Participants

We included all patients who received uncomplicated DMEK and for whom preoperative and postoperative AS-OCT data and visual acuity were available for analysis. Patients were excluded if no preoperative and postoperative AS-OCT scans were available for analysis, or if the first AS-OCT scan was performed more than 10 days after surgery. Patients who experienced intraoperative and/or early postoperative complications (eg, prolonged pupillary block, air bubble dislocation, upside-down grafts, complete graft detachment with free-floating graft in the anterior chamber, and folded grafts), for whom a different clinical and/or surgical approach was required, were also excluded.

### Data Measurement

Demographic data and clinical data of included patients were extracted from electronic patient records. Recorded clinical data included preoperative BCVA, indication for DMEK, date of surgery, preoperative lens status, whether a combined (Phaco-DMEK) procedure was performed, surgical time, graft type (surgeon-stripped or preloaded), donor age and ECD, need for rebubbling and date of rebubbling, final follow-up date, and final BCVA. Whenever available, BCVA was also recorded for each follow-up visit after surgery.

Anterior segment optical coherence tomography data for each patient were extracted from the available instrument at each clinic. The last preoperative and first postoperative AS-OCT scans available for each patient were analyzed. Central corneal thickness (CCT) values before and after DMEK were recorded.

**FIGURE 1.**
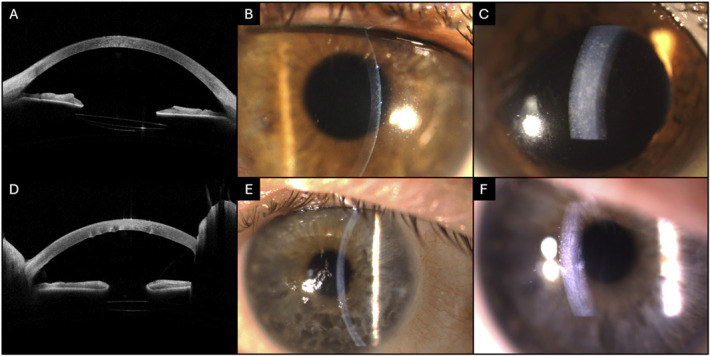
Preoperative PSR in pseudophakic patients with endothelial decompensation undergoing DMEK. A, the AS-OCT scan shows diffuse central corneal thickening with no pre-PSR. B, C, narrow and wide beam slit-lamp photography of the same patient shows the presence of corneal guttae and corneal edema but no pre-PSR. D, this AS-OCT scan shows the presence of pre-PSR and bullous keratopathy in a different patient. E, F, narrow and wide beam slit-lamp photography of the same patient. Pre-PSR are visible on slit-lamp examination, particularly with the use of a broader, tilted beam.

Posterior stromal ripples, defined as an irregularity in the posterior corneal profile that assumes the shape of a ripple,^13^ were identified by 2 cornea specialists (M.V. and M.A.). OCT scans were independently marked for the presence or absence of PSR. Disagreements were adjudicated by a senior specialist (V.R.). Explicative cases of PSR are illustrated in Figure 1.

### Variables and Outcomes

The primary outcome was the difference in longitudinal trends of visual acuity recovery after DMEK between eyes with and without pre-PSR.

Secondary outcomes included the distribution of preoperative and final values of BCVA and CCT, the estimated frequency of rebubbling, and measures of proportional relative risk of rebubbling according to the presence of preoperative and postoperative PSR.

### Statistical Analysis

Descriptive data were summarized using the mean (SD), median (interquartile range), and number (percentages) where appropriate.

Χ^2^ tests, Fisher tests, *t* tests, and Kruskal–Wallis tests were used as appropriate to compare demographic and clinical data among eyes with and without pre-PSR.

Predictions from a longitudinal generalized additive model, including visual acuity data from all eyes up to 12  months of follow-up regardless of whether they completed the follow-up, were used to visualize and compare visual acuity trends among eyes with and without pre-PSR, with an interaction between pre-PSR and time from surgery as the main predictor variable for the comparison. The model also included parametric terms to adjust for age and whether a combined surgery was performed (Phaco-DMEK) and a smooth random term to model individual trends.

A Cox proportional hazard regression model was used to estimate the hazard ratio (HR) for rebubbling according to the presence of preoperative and postoperative PSR. Only eyes that underwent DMEK with surgeon-stripped tissues were used to estimate the HR of rebubbling, to avoid confounding effects due to higher rates of rebubbling in preloaded tissues.^15,16^ Estimated values of rebubbling frequency were also obtained from the model. A Kaplan–Meier function was used to plot survival curves.

A *P* value less than 0.05 was considered statistically significant. All analyses were conducted using R software version 4.2.2 (R Project for Statistical Computing, Vienna, Austria).

## RESULTS

Electronic records of a total of 86 DMEKs performed between May 2021 and September 2023 at participating centers were reviewed. 15 cases were excluded from the analysis because of incomplete clinical data, for a total of 71 eyes from 66 patients.

### Demographic and Clinical Data

**FIGURE 2.**
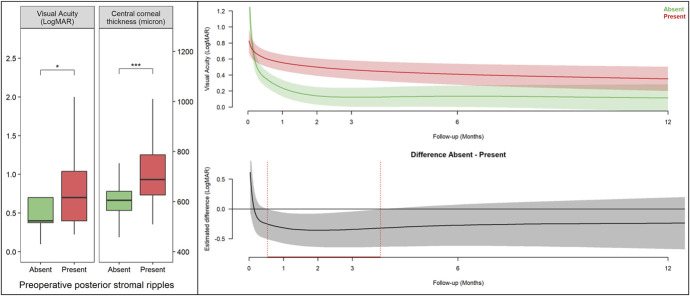
Left panel, differences in baseline values of visual acuity and CCT according to presence of preoperative PSR. Brackets indicate pairwise comparisons with *t* tests. *P < 0.05; **P < 0.01; ****P < 0.0001. Top right panel, mean estimated visual acuity trends over 12 months after surgery according to the presence of preoperative PSR. Bottom right panel, estimated difference in visual acuity change between eyes with no PSR and eyes exhibiting preoperative PSR. The gray shaded area in the bottom panel represents the 95% CI of the visual acuity trend estimate while the red dotted line represents the window of time in which the 95% CI does not cross zero (day 17–114). Visual acuity recovery was faster in patients with no preoperative ripples, but no significant differences could be identified after day 114. Predictions were made from a generalized additive model with interaction of time and presence of preoperative ripples as the main predictor.

Demographic data and clinical features of included patients are summarized in Table 1. Patients with no pre-PSR were on average significantly younger than patients who presented with pre-PSR [mean (SD), 66.3 (8.8) vs. 74.9 (8.9), *P* <0.001]. No significant difference could be identified in gender distribution, proportion of phakic eyes and combined surgery, indication for DMEK, type of graft, donor age and ECD, or surgical time. The presence of pre-PSR was associated with lower preoperative visual acuity [mean (SD), 0.6 (0.5) vs. 0.9 (0.6) LogMAR, *P* = 0.02] and higher corneal thickness [mean (SD), 613 (73.8) vs. 715.7 (129.6) micron, *P* <0.001] compared with eyes that showed no pre-PSR. The effect of pre-PSR on baseline BCVA and CCT is presented in Figure 2.

**TABLE 1.** Demographic and Clinical Data of Included Patients According to Presence of Preoperative PSR

	Absent	Present	*P*
Eyes, n	38	33	
Patients, n	35	31	
Females, n (%)	24 (68.6)	17 (54.8)	0.37
Age, mean (SD)	66.3 (8.8)	74.9 (8.9)	**<0.001**
Phakic eyes, n (%)	7 (18.4)	6 (18.2)	1
Combined Phaco-DMEK, n (%)	6 (15.8)	5 (15.2)	1
Presence of glaucoma drainage device, n (%)	2 (5.3)	1 (3)	1
Preoperative IOP, mean (SD)	13.3 (4.2)	9.6 (2.9)	0.26
Indication			0.32
FED	27 (71.1%)	20 (60.6%)	
PBK	8 (21.1%)	8 (24.2%)	
Graft failure	3 (7.9%)	2 (6.1%)	
Other*	0 (0%)	3 (9.1%)	
Baseline			
BCVA-LogMAR, mean (SD)	0.6 (0.5)	0.9 (0.6)	**0.02**
CCT-microns, mean (SD)	613 (73.8)	715.7 (129.6)	**< 0.001**
Graft and surgery data			
Graft type, n (%)			1
Surgeon-stripped	26 (68.4)	22 (66.7)	
Preloaded	12 (31.6)	11 (33.3)	
Donor age, mean (SD)	68.9 (7.7)	70.6 (9.1)	0.45
Donor ECD, mean (SD)	2662.2 (216.5)	2643.8 (200)	0.71
Surgical time-minutes, mean (SD)	66 (19.7)	63 (18.8)	0.61
Follow-up			
Days after surgery, median (IQR)	1 (1, 9)	2 (1, 7)	0.85
CCT-microns, mean (SD)	640.9 (152.3)	660.8 (109.8)	0.53
Postoperative stromal ripples, n (%)			0.81
Absent	24 (63.2)	19 (57.6)	
Present	14 (36.8)	14 (42.4)	
Rebubbling, n (%)			0.74
Yes	8 (21.1)	9 (27.3)	
No	30 (78.9)	24 (72.7)	
Days to rebubbling, median (IQR)	8 (6.8, 12.2)	7 (5, 11)	0.44
Final BCVA-LogMAR, mean (SD)	0.1 (0.2)	0.3 (0.3)	**0.02**
Final follow-up, mean (SD)	157.6 (127)	112.5 (128.5)	0.23

* Other indications include HSV endotheliitis, endothelial decompensation after iris cyst removal, and iridocorneal endothelial syndrome (n = 1 each).

BCVA, best corrected visual acuity; IOP, intraocular pressure; IQR, interquartile range; n, number; PBK, pseudophakic bullous keratopathy. Significant comparisons (*P* < 0.05) are highlighted in bold.

Although visual acuity improved in all groups after surgery, eyes with pre-PSR attained lower final visual acuity compared with eyes without preoperative PSR at the last follow-up [mean (SD), 0.1 (0.2) vs. 0.3 (0.3) LogMAR, *P* = 0.02]. CCT values were not significantly different after surgery.

No significant differences in the crude rate of rebubbling could be attributed to the presence of pre-PSR.

### Preoperative PSR and Visual Acuity Recovery Rate

Visual acuity trends after DMEK are illustrated in Figure 2. Longitudinal visual acuity data have been used to generate predictions of visual acuity trends up to 12 months after surgery (top right panel). Significant estimated differences between groups are highlighted in red in Figure 2 (bottom right panel). Visual acuity recovery was faster in the first months after surgery in patients who exhibited no pre-PSR, with a window of significant estimated difference in visual acuity, compared with eyes with pre-PSR that extended from day 17 to day 114.

### Proportional HRs of Rebubbling

A total of 48 eyes that received surgeon-stripped DMEK grafts were included in the analysis. Preloaded tissues (n = 23) have been shown to exhibit higher detachment rates^15,16^ and were, therefore, excluded to avoid overestimation of the HRs of rebubbling.

**FIGURE 3.**
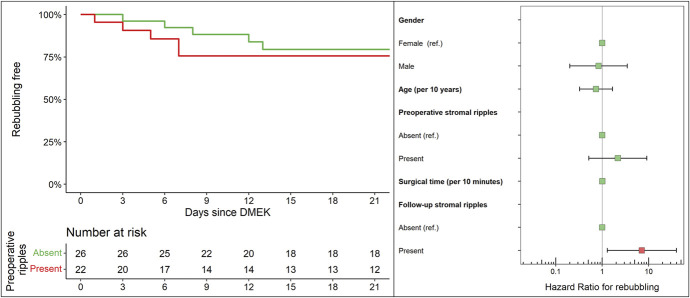
Left panel, Kaplan–Meier estimates and number of eyes at risk of needing rebubbling according to presence of preoperative PSR. Right panel, hazard ratios for rebubbling estimated from a Cox proportional hazard model. The presence of PSR was associated with a trend toward higher risks of rebubbling, both before and after surgery, although only the presence of postoperative PSR significantly increased the risk of rebubbling.

Kaplan–Meier estimates of rebubbling rates are presented in Figure 3. Cox proportional HRs showed no significant difference in relative risk due to the presence of preoperative PSR, age, sex, and surgical time (all *P* > 0.05). Postoperative PSR were instead associated with a significant increase in the risk of rebubbling [HR (95% CI), 7.1 (1.3, 39.5), *P* = 0.02]. These findings are presented in Figure 3. HRs for all covariates are provided in Table 2. The frequency of rebubbling ranged from 8.2%–20.5% for eyes without preoperative or postoperative ripples to 24.4%–36.4% in eyes that showed either preoperative or postoperative PSR, respectively.

**TABLE 2.** Estimated Hazard Ratios for Rebubbling

	Hazard Ratio (95% CI)	*P*
Sex		
Female (ref.)		
Male	0.8 (0.2, 3.5)	0.8
Age (per 10 years)	0.7 (0.3, 1.7)	0.46
Preoperative stromal ripples		
Absent (ref.)		
Present	2.2 (0.5, 9.2)	0.62
Surgical time (per 10 minutes)	1 (1, 1)	0.71
Postoperative stromal ripples		
Absent (ref.)		
Present	7.1 (1.3, 39.5)	**0.02**

Significant HR (*P* < 0.05) are highlighted in bold.

## DISCUSSION

In this study, we found a significant association between the presence of pre-PSR and delayed visual acuity recovery after DMEK, particularly during the first 114 days (∼3.5 months) postoperatively. Eyes that exhibited pre-PSR also attained a lower final visual acuity compared with eyes without pre-PSR. Finally, pre-PSR were associated with higher preoperative CCT values and lower preoperative visual acuity.

Considering the impact of PSR on postoperative visual acuity after DMEK, it could be speculated that PSR might be associated with irreversible ultrastructural changes that negatively influence postoperative surgical outcomes or that PSR become manifest when such changes occur. In particular, the effect on the rate of visual recovery and final visual acuity that we found in this study could be explained if we consider PSR as a possible marker manifesting in the final stages of corneal endothelial dysfunction, when damage to the corneal lamellae that cannot be reverted or mitigated by DMEK has already happened.

Previous studies have explored the role of morphological preoperative corneal changes (collagen disorganization, keratocyte degeneration, anterior stromal scars, and subepithelial fibrosis) on the postoperative BCVA.^9,10,17^

Hecker et al^9^ found a reduction of anterior stromal keratocytes in corneas affected by endothelial dysfunction compared with healthy ones. Specifically, using histology and confocal microscopy, they demonstrated that a reduction of keratocytes by 54% to 63% in the anterior 10% of the stroma contributed to persistent anterior stromal changes that degraded visual acuity after endothelial keratoplasty. In 2013, Patel and McLaren demonstrated a correlation between the presence of abnormal subepithelial fibroblasts in corneas with Fuchs endothelial dystrophy and lower visual outcomes after surgery.^10^ Finally, Morishige et al^17^ found fibroblastic cells in the anterior stroma in patients with a preoperative duration of stromal edema of more than 12 months, evaluated by in vivo laser confocal microscopy.

Multiple studies have also shown that prolonged Fuchs endothelial dystrophy (FED) results in an elevation of corneal backscatter.^18–21^ Corneal haze is responsible for a pathological scattering of light and represents an indication of corneal clarity and optical quality. The processes causing corneal haze often contribute to forward scatter and backscatter of light and high-order aberrations, both of which degrade visual acuity. McLaren et al found a strong correlation between the backscatter from the anterior 120 μm of the cornea—measured with a Pentacam Scheimpflug camera and a corneal confocal microscope—and the severity of FED.^22^ A correlation of FED severity to the backscatter from the mid-cornea and the posterior 60 μm of the cornea was instead found only with the Pentacam densitometry analysis. Recently, the presence of a “fibrillar layer” of collagen-rich extracellular matrix in eyes with advanced FED has been identified through immunohistochemical studies and its imaging correlates described with Scheimpflug tomography.^23^ Similar to preoperative posterior stromal ripples, this finding seems to correlate with disease severity and possibly to influence visual recovery. Although we could not verify the influence of PSR on the corneal backscatter, we can imagine that higher degrees of PSR could potentially correlate with more severe optical disturbance. Additional studies will be required to estimate the influence of PSR on the densitometry of the posterior cornea and high-order aberrations.

For what concerns the risk of rebubbling, we have demonstrated in a previous study an association between postoperative PSR and a higher risk of graft detachment requiring rebubbling.^13^ Several preoperative and postoperative factors predisposing to graft detachment and rebubbling after DMEK have been identified.^3–5,12,13,15,16,24–28^ In this study, we found that both preoperative and postoperative PSR were associated with a trend toward higher risks of rebubbling. However, only the presence of postoperative PSR significantly elevated the risk of rebubbling, in accordance with the findings by Coco et al.^13^

In this study, we also found a correlation between the presence of pre-PSR and higher preoperative CCT. Several studies have explored the influence of preoperative CCT on DMEK outcomes.^3,24,29–31^ Brockmann et al^3^ demonstrated that preoperative corneal thickness exceeding 625 μm was significantly associated with an advanced stage of FED and lower postoperative BCVA. Conversely, other studies failed to find this correlation, hypothesizing that only postoperative CCT reflects the corneal endothelial graft function.^4,29,31,32^ Schrittenlocher et al^29^ demonstrated that lower preoperative visual acuity was linked to corneal ultrastructural changes rather than pachymetry because isolated corneal edema is remedied by surgery. In addition, it is also difficult to designate a cutoff for CCT, as CCT varies for each patient. Age, ethnicity, and genetic factors have a significant influence during ocular growth, and therefore, increased corneal thickness does not always mean an edematous cornea.^33,34^ Unlike CCT, pre-PSR are a categorical imaging biomarker, and their presence can help gauge visual recovery and identify eyes at risk of reduced postoperative BVCA.

In cases of endothelial failure, the level of corneal hydration increases, resulting in a proportional thickening. The binding of water by stromal proteoglycans associated with collagen generates a potent inward pressure gradient known as swelling pressure.^35^ Stromal swelling is attributed to the imbibition of water between fibrils rather than the swelling of collagen fibrils. Histologically, ultrastructurally, and biochemically, distinctions exist between the anterior third and posterior two-thirds of the cornea.^36^ The anterior stroma contains less water compared with the posterior stroma. This variation is attributed to a higher concentration of glucose in the posterior stroma and an uneven distribution of proteoglycans throughout the cornea.^37^ Dermatan sulfate is predominantly located in the anterior portion of the cornea, whereas the posterior part contains more keratan sulfate. Dermatan sulfate exhibits superior water retention but lower water absorption capacity while keratan sulfate demonstrates the opposite characteristics. This discrepancy clarifies why corneal swelling is predominantly observed in the posterior cornea and rapidly resolves when the endothelial pump function recommences following transient damage.^35,37,38^ Thus, PSR might appear when the hydration level of the posterior stroma has reached its maximum and the posterior, keratan-sulfate rich portion of the cornea swells.

Supported by these pathophysiological mechanisms, in this study and in our previous work,^13^ we presented compelling evidence to consider PSR a strong prognostic factor of outcomes after DMEK. PSR also seem to be an independent prognostic biomarker: surgical indication, previous intraocular surgeries, presence of filtration devices, and preoperative intraocular pressure in this study were all comparable between patients with and without PSR, supporting the idea that PSR do not represent a surrogate marker of underlying pathology but are in fact an indicator of marked endothelial dysfunction, independent of ocular comorbidities and CCT. Thus, PSR can be used as an imaging biomarker to estimate visual recovery rates preoperatively and risk of rebubbling postoperatively and probably represent a more reliable marker than absolute corneal thickness. In fact, corneal thickness can vary significantly among healthy individuals, with variations that may range from approximately 400 to 600 μm.^39–41^ Establishing a universal cutoff for what is considered normal or abnormal can be challenging for several reasons: individual variation, ethnic and genetic differences, measurement techniques, time of day, and age. On the contrary, PSR could represent a one-size-fits-all approach. When each individual cornea reaches its maximum posterior hydration, ripples appear on the endothelial side. This approach may have certain advantages, given its simplicity and efficiency: It reduces the need for customization or individual tailoring, streamlining procedures, and decision making; it promotes consistency across different clinical situations; and it facilitates communication with the patient by making the results more predictable.

Limitations of this study are mainly attributable to its design. Given the retrospective nature of the study, no control over the distribution of preoperative characteristics among DMEK patients with and without pre-PSR was possible. Since we collected real-world clinical data, patients could have different follow-up lengths that could have affected the calculation of crude visual acuity outcomes. To account as much as possible for this issue, we used a generalized additive model to estimate visual acuity recovery rates, making estimations based on all available data up to 12 months after surgery. Eyes included in this study were imaged before and after DMEK with AS-OCT, but other imaging modalities that might be altered in Fuchs endothelial dystrophy such as Scheimpflug tomography were not available in all cases, and comparisons with such modalities will, therefore, have to be explored in the future works. Finally, treatment decisions in routine clinical practice were made according to the treating physician's decision in consultation with the patient. This may have resulted in differences in outcomes across the centers; however, this was mitigated by including patients with and without pre-PSR from each center.

Strengths of this study include its multicenter nature, which increases the generalizability of our results, with similar instrumentation, surgical technique, surgical experience, and follow-up pattern across centers.

In conclusion, we can hypothesize that preoperative PSR might be correlated with long-term morphological corneal changes and we have shown that preoperative PSR contribute to a slower visual recovery and worse final postoperative BCVA. We confirmed that postoperative PSR are associated with weaker graft adhesion, higher risk of graft detachment, and elevated risk of rebubbling. PSR represent a valuable imaging biomarker before and after DMEK that can help cornea specialists stratify patients and identify patients at higher risk of suboptimal postoperative outcomes.
